# Older age bipolar disorder

**DOI:** 10.1097/YCO.0000000000000883

**Published:** 2023-07-17

**Authors:** Alexandra J.M. Beunders, Melis Orhan, Annemiek Dols

**Affiliations:** a GGZ inGeest Specialized Mental Healthcare; b Amsterdam UMC Location Vrije Universiteit Amsterdam, Psychiatry; c Amsterdam Public Health Research Institute, Mental Health, Amsterdam; d Institute of Clinical Psychology, Leiden University, Leiden; e Department of Psychiatry, UMC Utrecht Brain Center, University Medical Center Utrecht, Utrecht, The Netherlands

**Keywords:** bipolar, cognition, comorbidities, functioning, older

## Abstract

Older age bipolar disorder (OABD) refers to patients with bipolar disorder aged 50 years and over. There is a paucity of evidence-based guidelines specific to OABD, but in recent years, several studies have been published on OABD. The current review synthesizes previous literature (up to January 1, 2021) as well as most recent literature on OABD (since January 1, 2021).

**Recent findings:**

This review covers the following themes: diagnosis and specifiers, clinical course, psychosocial functioning, cognition, physical comorbidities, and pharmacotherapy. On the basis of the latest data, specific clinical recommendations are proposed for each theme.

**Summary:**

OABD forms a more complex subgroup of bipolar disorder, with an increased risk of cognitive deficits, physical comorbidities, impaired psychosocial functioning, and premature death. The distinctions between BD-I and BD-II and between EOBD and LOBD do not clinically represent relevant subtypes for OABD patients. Mental healthcare professionals should treat all OABD patients with an integrative care model that takes into account cognitive and physical comorbidities and that contains elements aimed at improvement of psychosocial functioning and quality of life. Older age itself should not be a reason to withhold lithium treatment. Future research should collect data on essential data domains using validated measurement scales.

## INTRODUCTION

Older age bipolar disorder (OABD) refers to patients with bipolar disorder aged 50 years and over. The International Society for Bipolar Disorders (ISBD) Task Force on OABD has recommended this age cut off [1] with the argument that bipolar disorder is a severe mental illness with a reduced life-expectancy of approximately 10–20 years [2]. Moreover, biological age is preceded by chronological age in bipolar disorder [3], leading to premature aging.

OABD forms a special more complex subgroup of bipolar disorder, with prevalent cognitive deficits [4,5], increased risk of dementia [6], impaired psychosocial functioning [7], frequent physical comorbidities [8], and premature death [2,9]. There are several factors associated with aging that also negatively influence outcome, such as a decreasing social network size, loss of support from friends and family members, lifestyle choices, reduced mobility, increased presence of poor physical health, and other aging-related issues [7]. Although OABD does not resolve or “burn out,” epidemiologic studies indicate that bipolar disorder affects 0.5–1.0% of older adults, which is lower than the prevalence of 1.4% reported in patients aged 18–44 years [10]. The absolute number of individuals with OABD is expected to rise due to the aging of the population.

**FIGURE 1:**
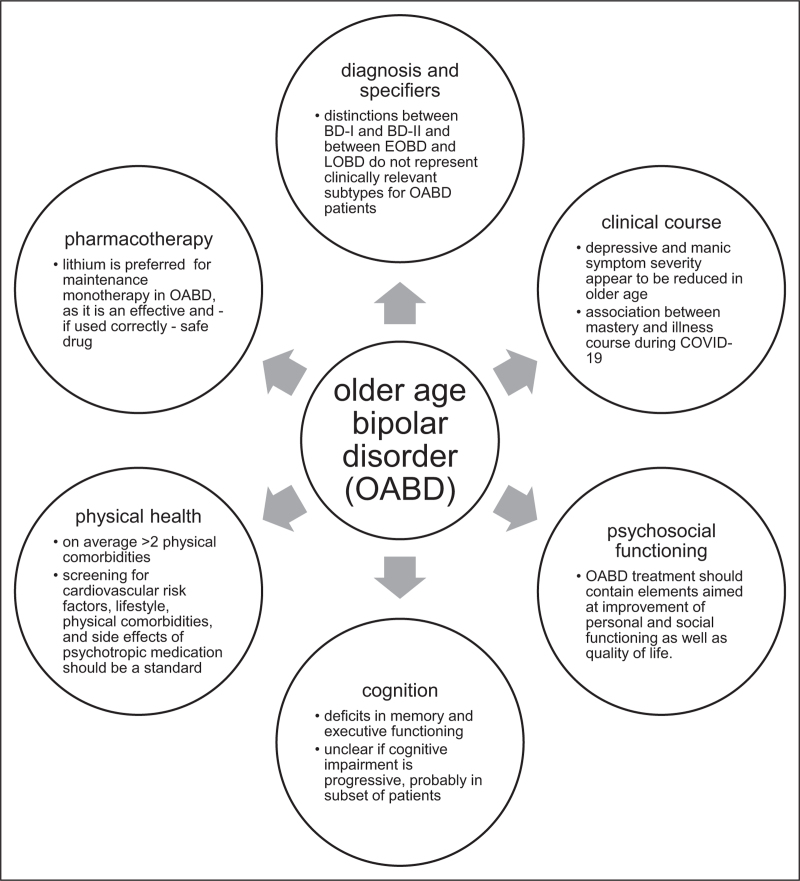
Summary of recommendations for treatment of patients with older age bipolar disorder based on the latest literature, as discussed in the current review.

A literature review and expert consensus report [1] as well as a survey of guideline recommendations for OABD [11] by the International Society for Bipolar Disorders (ISBD) taskforce for OABD indicated that there is a paucity of evidence-based guidelines specific to OABD. To fill this gap, the Global Aging & Geriatric Experiments in Bipolar Disorder (GAGE-BD) project was started in 2018. Funded by Bowden Massey Strategic Research Initiative in Bipolar Disorder Award [12], today, over 20 international investigators have joined with data of at least 2200 OABD patients [13^▪^]. In recent years, also other research groups have performed research in specific OABD populations. The current review synthesizes what was already known (literature up to January 1, 2021) as well as most recent literature on OABD (since January 1, 2021). This review focuses on themes with high clinical relevance for OABD: diagnosis and specifiers, clinical course, psychosocial functioning, cognition, physical comorbidities, and pharmacotherapy (Fig. 1). 

**Box 1:**
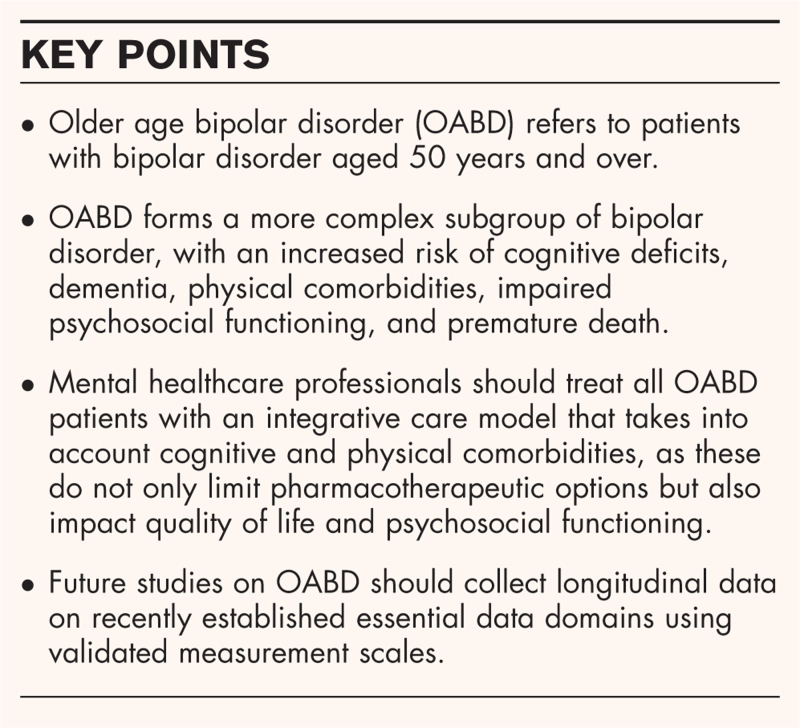
no caption available

## DIAGNOSIS AND SPECIFIERS

There is a lot of variability within the diagnostic category of bipolar disorder, also in older adults. In clinical practice, clinicians find it difficult to estimate the severity of disease, and consequently, the preferred amount of monitoring and treatment for an individual patient. There is a need for subtyping (profiling) OABD patients to reduce this heterogeneity and to improve personalized treatment. Markers of illness severity that have previously been suggested for bipolar disorder patients of all ages are (among others): bipolar disorder subtype I [14], presence of psychotic symptoms [15], childhood maltreatment [16], predominant polarity, episode density [17], and presence of neuroinflammation [18,19].

A common way of profiling is to differentiate between bipolar disorder subtype I and II. BD-II is often considered a “milder” form of BD-I, but observed clinical differences in severity may also be due to the DSM-5 definition itself [20]. A second specifier is the distinction of early-onset bipolar disorder (EOBD) and late-onset bipolar disorder (LOBD). In the past, subtyping by age of onset seemed of clinical relevance, as LOBD was often viewed as an expression of preclinical dementia with a poor response to mood stabilizers, whereas EOBD was viewed as more classic bipolar disorder with a positive family history for mood disorders [21]. Using the first wave of the Dutch Older Bipolar (DOBi) cohort (*N* = 101 OABD patients), Dols *et al.*[22] found that physical and psychiatric comorbidities were not different between BD-I (*N* = 57) and BD-II (*N* = 47) or EOBD (*N* = 84) and LOBD (*N* = 17).

### What is new in older age bipolar disorder?

Regarding age of onset, the GAGE-BD project compared EOBD and LOBD patients [23^▪^]. The authors reported that EOBD patients were not different in terms of severity of depressive and manic symptoms and functioning than LOBD patients. A second GAGE-BD report found that symptom mixity (i.e., both depressive and manic symptoms at the same time) is common in OABD patients and that mixed features are associated with worse everyday function [24]. Yet another GAGE-BD report compared BD-I and BD-II subtypes in older age [25^▪^]. BD-I and BD-II older-aged patients appeared similar in general functioning, cognitive performance, and somatic burden. A review on neuroimaging suggested that in a subset of OABD patients, aging is accompanied by a pronounced loss of gray and white matter, which may contribute to adverse outcomes [26].

### Clinical recommendations

The distinctions between BD-I and BD-II and between EOBD and LOBD do not seem to represent clinically relevant subtypes for OABD patients. Future research should examine potential other disease specifiers in OABD populations [27^▪^], such as history of psychotic features, predominant polarity, episode density, and childhood maltreatment.

## CLINICAL COURSE

The clinical course of OABD is highly variable: the illness remains stable in some patients but seems to progress in others. Illness progression can result in treatment resistance, a shorter time between mood episodes, and decreased cognitive functioning [28,29]. In a Dutch naturalistic cohort study in OABD (DOBi, *N* = 64), 37.5% of patients reported at least one recurrent episode within 3-year follow-up, mainly depression. Life events, somatic illness, and use of lithium were not associated with recurrence during 3-year follow-up [30].

According to the kindling-hypothesis [31,32], symptom-free intervals shorten over time in bipolar disorder. This hypothesis states that with every episode, the risk of recurrence increases. However, it seems that this cycle acceleration only holds true for the first three episodes. After these, the acceleration decreases and the duration of the symptom-free episodes becomes more stable [33].

Staging models provide a way to improve diagnostic precision by categorizing patients with similar illness progression. Two staging models have received the most attention in bipolar disorder; “Model A” [34], which is based on the number and recurrence of mood episodes and “Model B” [35], which is based on the level of inter-episodic functioning.

### What is new in older age bipolar disorder?

Results from GAGE-BD suggest that both depressive and manic symptom severity appear to be reduced in older age [13^▪^]. In a recent DOBi study (*N* = 64), social, psychological, and cognitive factors were not related to recurrence during 3-year follow-up [36]. However, OABD patients with recurrence were younger, more often female, and less likely to have children. The COVID-19 pandemic offered the opportunity to study predictors for recurrence during a collective negative life event within the DOBi cohort [37]. Not having children, more feelings of loneliness, passive coping style, and higher neuroticism were found to be associated with more psychiatric symptoms during COVID-19. A higher sense of mastery was protective initially, but over the course of 6 months, a higher sense of mastery was associated with a greater increase in mood symptoms. However, participants with higher mastery still reported less psychiatric symptoms than participants with a lower sense of mastery [38]. Furthermore, earlier mentioned staging models A and B were explored in the first-ever study on staging in OABD patients [39]. For model A, based on the number and recurrence of mood episodes, a higher stage and thus less favorable course of illness was associated with childhood abuse, longer illness duration, and higher episode density. Model B, based on inter-episodic functioning, may be less suitable for OABD as currently operationalized or may measure different aspects of illness progression [39].

### Clinical recommendations

The observed association between mastery and the course of bipolar disorder during COVID-19 stresses the need to develop psychotherapeutic strategies to prevent recurrence in OABD.

## PSYCHOSOCIAL FUNCTIONING

Older age has been associated with lower psychosocial functioning in bipolar disorder [40]. Previous OABD studies demonstrated that psychosocial functioning was low in a number of areas, such as autonomy, independence, economic management, occupational performance, and interpersonal relationships [41,42]. Assessment methods for daily functioning in OABD are sparse, as many commonly used scales are not specifically designed for OABD. However, the Functioning Assessment Short Test for Older adult (FAST-O) offers the opportunity to assess the current level of functioning in the OABD population [43].

### What is new in older age bipolar disorder?

Findings from the GAGE-BD project [12] suggested that greater severity of depressive symptoms in bipolar disorder was associated with worse functioning in OABD [44]. Greater somatic burden was not associated with reduced functioning in the full GAGE-BD sample [13^▪^]. In a small DOBi study (*N* = 60), nonaffective cognitive impairment was associated with worse social functioning, but this was not the case for affective cognition [45].

### Clinical recommendations

As recovery in OABD is more than reducing clinical symptoms, OABD treatment should also contain elements aimed at improvement of personal and social functioning as well as quality of life. As functioning contains different aspects, it is important to include multiple areas of functioning in assessment and monitoring. Therefore, it is essential to use assessment instruments validated in OABD, for instance the FAST-O [43].

## COGNITION

Cognitive dysfunction occurs frequently in OABD. It is estimated that 40–50% of OABD experience cognitive dysfunction during euthymia. It is therefore among the most persistent symptoms in OABD [46,47]. Cognitive dysfunction in OABD and its course are highly heterogeneous. A higher dementia prevalence is found in bipolar disorder compared with healthy peers and other psychiatric populations [48–51]. In middle-aged bipolar disorder patients, several studies have identified three different groups based on their cognitive profile, varying from a severely impaired cognitive functioning to preserved cognitive performance [52–55]. Regarding the older population, some studies suggest a more severe decline of cognitive functioning in OABD compared with healthy older adults, whereas others refute a progressive course in OABD [4,56,57]. Different hypotheses have been postulated on cognitive impairment in bipolar disorder. The cognitive reserve hypothesis states that patients with a high IQ, education level, or occupation are less likely to develop dementia. In bipolar disorder patients, this reserve is believed to be decreased due to vascular burden. Therefore, the hazard that a bipolar disorder patient develops dementia is thought to increase over time [57]. On the contrary, the neuroprogression hypothesis states that every mood episode, especially with manic and psychotic symptoms, is toxic for the brain [58–60]. In this light, cognitive deficits and mild structural differences are believed to be the result of accelerated aging in OABD [61].

### What is new in older age bipolar disorder?

Although heterogeneity in cognitive performance in bipolar disorder remains at an older age [62^▪^], a recent meta-analysis confirmed that several cognitive domains are often affected in OABD, even during euthymic states. However, dysfunction was mostly observed in the memory domain and no significant correlations were found between clinical illness or demographic variables [62^▪^]. In a recent cross-sectional study (*N* = 432 bipolar disorder patients), older age was associated with a selective cognitive decline in bipolar disorder in attention when compared with age-matched healthy controls [63]. OABD patients show a more severely impaired cognitive profile when compared with older patients with unipolar depression or healthy controls [64,65]. A recent DOBi study suggests that cognitive performance is multifactorial, as cardiovascular risk, benzodiazepine use, number of episodes, late-onset, and five or more psychiatric admissions were associated with cognitive performance [66]. A cluster analysis also identified three groups in older bipolar disorder patients: a preserved group, a group with mild deficits in all cognitive domains, and a group with moderate to severe cognitive impairments [67]. A meta-analysis showed that bipolar disorder was associated with an increased risk of subsequent all-cause dementia [68].

### Clinical recommendations

As OABD patients are vulnerable for cognitive deficits, clinicians need to monitor cognitive function throughout treatment. Risk factors need to be addressed in treatment programs in order to prevent further cognitive decline.

## PHYSICAL COMORBIDITIES

In bipolar disorder of all ages, physical burden is high [69,70]. Physical diseases are much more common in bipolar disorder patients than in healthy controls [71]. In particular, migraine, asthma, hyperlipidemia, hypertension, thyroid disease, osteoarthritis, epilepsy, renal disease, influenza or pneumonia, COPD, diabetes, cardiovascular disease, and stroke are more prevalent [69,71,72]. The mortality ratio of individuals with bipolar disorder is two to three times higher than in the general population [71,73] and this mortality gap has widened in the last 20 years [73–75]. The life expectancy of bipolar disorder patients is shortened with 9–20 years [2,76], which is mainly due to general physical diseases and particularly cardiovascular diseases (CVDs) [77,78]. In bipolar disorder, CVD also appear at a younger age than in the general population [79–81], suggesting accelerated aging [3]. Possible causes of the high physical burden are an unhealthy lifestyle (smoking, alcohol use, nutrition, physical exercise, stress, sleep), decreased access to (high quality) medical care, adverse effects of pharmacotherapy, severity of bipolar disorder, and genetic factors [70,71]. High physical burden has been associated with a more severe illness course, reduced general and cognitive functioning, and possibly a poorer treatment response and rapid cycling [66,82,83].

### What is new in older age bipolar disorder?

A recent systematic review provides tentative evidence that some physical comorbidities are elevated in OABD, in particular cardiovascular disease and some forms of cancer [84]. The Dutch naturalistic DOBi study (*N* = 101) found one or more physical comorbidities in 62% with an average of 1.2 out of eight diseases [85]. Compared with the older general population (1.6 diseases), the prevalence was lower in the OABD group at baseline, which may be due to a “survivor effect”: the hypothesis that patients with the highest physical burden are not included in OABD studies as they have already died. Still, a faster accumulation of chronic physical diseases was observed in the OABD group during 3-year follow-up [85]. Recently, the GAGE-BD project reported extensive physical burden (mean 2.4 diseases) in a global sample of 1377 older individuals [13^▪^]. Within the OABD group, women were more affected than men [86^▪^]. Compared with older Australian men from the general population, physical comorbidities were more prevalent in OABD men [86^▪^].

### Clinical recommendations

Systematic screening for cardiovascular risk factors and assessment of lifestyle, physical comorbidities, and side effects of psychotropic medication should be a standard in bipolar disorder patients of all ages. Psychiatric clinicians should collaborate closely with general practitioners and other medical specialists.

## PHARMACOTHERAPY

When prescribing medication to older adults, it is important to take into account presence of physical comorbidities and cognitive impairment. Also, due to altered pharmacokinetics and pharmacodynamics, older patients are more vulnerable for side effects and drug interactions. In bipolar disorder populations, antipsychotics have been associated with an increased risk for many physical diseases, including respiratory disease, cardiovascular disease, thyroid disease, gastro-intestinal, musculoskeletal, and renal diseases [87]. Many drugs, among which olanzapine, quetiapine, and clozapine can cause severe weight gain [88]. Benzodiazepines and antipsychotics have been associated with cognitive impairment [66,89]. Research on pharmacological treatment of OABD is scarce, as older patients or patients with physical comorbidities are often excluded from drug research. However, open-label trials, naturalistic studies, and posthoc analyses of mixed-aged RCTs suggest that medications efficacious in younger adults will also be effective in older adults [90]. The 2018 CANMAT-ISBD guideline for bipolar disorder includes a special section on older patients [90]. The ISBD task force for OABD has published a Delphi study with specific recommendations on lithium use based on the opinion of 25 OABD experts [91]. The authors stated that lithium is the preferred choice for maintenance monotherapy in OABD, as it is an effective and – if used correctly – well tolerated drug, also in the older population [92,93]. However, special caution is required in order to prevent nephropathy and intoxication [90–93]. Dose reduction of lithium is often required in older patients, with recommended serum levels of 0.4–0.8 mmol/l for age 60–79 and 0.4–0.7 mmol/l for age 80+ [94].

### What is new in older age bipolar disorder?

A large observational aging cohort (UK Biobank, *N* = 501 461 individuals) reported that lithium users had a 4.6 times lower change of dying compared with users of other antipsychotic drugs, so lithium can be seen as a “geroprotective supplement” [95]. The GAGE-BD project analyzed lithium users vs. nonusers and found that OABD lithium users had less depressive symptoms, less rapid cycling, and fewer psychiatric and fewer cardiovascular comorbidities. On the contrary, lithium users had better cognitive functioning than nonusers [96^▪^]. A Dutch retrospective cohort study analyzed 135 older lithium users (median age 69) [97]. During follow-up, 49 (36.3%) patients discontinued lithium, but only a minority (*n* = 11; 8.1%) of the participants discontinued solely due to adverse effects. The GAGE-BD project also compared antipsychotic users with nonusers. This analysis showed that individuals with OABD on antipsychotics have more severe illness, more frequent hospitalizations, and are more often unemployed [98^▪^]. The ISBD OABD task force performed a systematic review on nutritional supplements in OABD. The authors provided low-quality evidence for associations between nutritional interventions (e.g., vitamin B12 and D) and improvement in affective, cognitive, and overall outcome in OABD [99].

### Clinical recommendations

New data confirm that older age itself should not be a reason to withhold lithium treatment. Clinicians should monitor serum levels and kidney function closely and should, in case of adverse effects, not stop lithium at once, but switch to an alternative drug with a more preferable side effect profile.

## CONCLUSION

OABD forms a more complex subgroup of bipolar disorder, with an increased risk of cognitive deficits, dementia, physical comorbidities, impaired psychosocial functioning, and premature death. Mental healthcare professionals should treat all OABD patients with an integrative care model that takes into account cognitive and physical comorbidities and that contains elements aimed at improvement of psychosocial functioning and quality of life. Within the OABD group, heterogeneity is high. Therefore, clinicians need to be aware of possible comorbidities, while at the same time trying not to lose sight of the individual patient. Future research is warranted to meet the needs of this special and expanding group. Researchers should collect longitudinal data on recently established essential data domains using validated measurement scales [27^▪^].

## Acknowledgements

*The authors thank the researchers of the Dutch Older Bipolar study (DOBi) and the GAGE-BD project for many fruitful collaborations.*

### Financial support and sponsorship

*Dr Dols was one of the initiators of the GAGE-BD project. This project was supported by the International Society for Bipolar Disorders (ISBD) Bowden Massey Strategic Research Initiative and made possible by logistical support from the ISBD.*

### Conflicts of interest

*Dr Dols is the Principal Investigator of the Dutch Older Bipolar study (DOBi) and one of the initiators of the GAGE-BD project. Dr Dols is chair of the ISBD taskforce for OABD. This publication's contents are solely the responsibility of the authors and do not necessarily represent the official views of ISBD. The ISBD is a 401c3 nonprofit organization whose mission is to foster international collaboration in education and research. For more information, visit*www.isbd.org.

## REFERENCES AND RECOMMENDED READING

Papers of particular interest, published within the annual period of review, have been highlighted as:

▪ of special interest
▪▪ of outstanding interest

## References

[1] Sajatovic M Strejilevich SA Gildengers AG. A report on older-age bipolar disorder from the International Society for Bipolar Disorders Task Force. Bipolar Disord 2015; 17:689–704. 26384588 10.1111/bdi.12331 PMC4623878

[2] Kessing LV Vradi E McIntyre RS Andersen PK. Causes of decreased life expectancy over the life span in bipolar disorder. J Affect Disord 2015; 180:142–147. 25909752 10.1016/j.jad.2015.03.027

[3] Rizzo LB Costa LG Mansur RB. The theory of bipolar disorder as an illness of accelerated aging: implications for clinical care and research. Neurosci Biobehav Rev 2014; 42:157–169. 24548785 10.1016/j.neubiorev.2014.02.004

[4] Schouws SN Comijs HC Dols A. Five-year follow-up of cognitive impairment in older adults with bipolar disorder. Bipolar Disord 2016; 18:148–154. 26961121 10.1111/bdi.12374

[5] Samame C Martino DJ Strejilevich SA. A quantitative review of neurocognition in euthymic late-life bipolar disorder. Bipolar Disord 2013; 15:633–644. 23651122 10.1111/bdi.12077

[6] Kessing LV Andersen PK. Does the risk of developing dementia increase with the number of episodes in patients with depressive disorder and in patients with bipolar disorder? J Neurol Neurosurg Psychiatry 2004; 75:1662–1666. 15548477 10.1136/jnnp.2003.031773 PMC1738846

[7] van Liempt S Dols A Schouws S. Comparison of social functioning in community-living older individuals with schizophrenia and bipolar disorder: a catchment area-based study. Int J Geriatr Psychiatry 2017; 32:532–538. 27121916 10.1002/gps.4490

[8] Lala SV Sajatovic M. Medical and psychiatric comorbidities among elderly individuals with bipolar disorder: a literature review. J Geriatr Psychiatry Neurol 2012; 25:20–25. 22467842 10.1177/0891988712436683

[9] Almeida OP McCaul K Hankey GJ. Risk of dementia and death in community-dwelling older men with bipolar disorder. Br J Psychiatry 2016; 209:121–126. 27482038 10.1192/bjp.bp.115.180059

[10] Kessler RC Berglund P Demler O. Lifetime prevalence and age-of-onset distributions of DSM-IV disorders in the National Comorbidity Survey Replication. Arch Gen Psychiatry 2005; 62:593–602. 15939837 10.1001/archpsyc.62.6.593

[11] Dols A Kessing LV Strejilevich SA. Do current national and international guidelines have specific recommendations for older adults with bipolar disorder? A brief report. Int J Geriatr Psychiatry 2016; 31:1295–1300. 27442023 10.1002/gps.4534

[12] Sajatovic M Eyler LT Rej S. The Global Aging & Geriatric Experiments in Bipolar Disorder Database (GAGE-BD) project: understanding older-age bipolar disorder by combining multiple datasets. Bipolar Disord 2019; 21:642–649. 31081573 10.1111/bdi.12795

[13▪] Sajatovic M Dols A Rej S. Bipolar symptoms, somatic burden, and functioning in older-age bipolar disorder: analyses from the Global Aging & Geriatric Experiments in Bipolar Disorder Database project. Bipolar Disord 2022; 24:195–206. 34314549 10.1111/bdi.13119 PMC8792096

[14] Serafini G Gonda X Aguglia A. Bipolar subtypes and their clinical correlates in a sample of 391 bipolar individuals. Psychiatry Res 2019; 281:112528. 31493714 10.1016/j.psychres.2019.112528

[15] Chakrabarti S Singh N. Psychotic symptoms in bipolar disorder and their impact on the illness: a systematic review. World J Psychiatry 2022; 12:1204–1232. 36186500 10.5498/wjp.v12.i9.1204 PMC9521535

[16] Agnew-Blais J Danese A. Childhood maltreatment and unfavourable clinical outcomes in bipolar disorder: a systematic review and meta-analysis. Lancet Psychiatry 2016; 3:342–349. 26873185 10.1016/S2215-0366(15)00544-1

[17] Garcia-Jimenez J Gutierrez-Rojas L Jimenez-Fernandez S. Features associated with depressive predominant polarity and early illness onset in patients with bipolar disorder. Front Psychiatry 2020; 11:584501. 33304285 10.3389/fpsyt.2020.584501 PMC7701086

[18] Knight EL Engeland CG Yocum AK. Heightened inflammation in bipolar disorder occurs independent of symptom severity and is explained by body mass index. Brain Behav Immun Health 2023; 29:100613. 37025250 10.1016/j.bbih.2023.100613 PMC10070374

[19] Wu X Chen Z Liao Y. Are serum levels of inflammatory markers associated with the severity of symptoms of bipolar disorder? Front Psychiatry 2022; 13:1063479. 36741577 10.3389/fpsyt.2022.1063479 PMC9894870

[20] Parker G Fletcher K McCraw S. Identifying antecedent and illness course variables differentiating bipolar I, bipolar II and unipolar disorders. J Affect Disord 2013; 148:202–209. 23265987 10.1016/j.jad.2012.11.061

[21] Dols A Beekman A. Older age bipolar disorder. Clin Geriatr Med 2020; 36:281–296. 32222302 10.1016/j.cger.2019.11.008

[22] Dols A Rhebergen D Beekman A. Psychiatric and medical comorbidities: results from a bipolar elderly cohort study. Am J Geriatr Psychiatry 2014; 22:1066–1074. 24495405 10.1016/j.jagp.2013.12.176

[23▪] Lavin P Buck G Almeida OP. Clinical correlates of late-onset versus early-onset bipolar disorder in a global sample of older adults. Int J Geriatr Psychiatry 2022; 37: [Epub ahead of print]. 10.1002/gps.5833 36317317

[24] Eyler LT Briggs FBS Dols A. Symptom severity mixity in older-age bipolar disorder: analyses from the Global Aging and Geriatric Experiments in Bipolar Disorder Database (GAGE-BD). Am J Geriatr Psychiatry 2022; 30:1096–1107. 35637088 10.1016/j.jagp.2022.03.007 PMC10280310

[25▪] Beunders AJM Klaus F Kok AAL. Bipolar I and bipolar II subtypes in older age: results from the Global Aging and Geriatric Experiments in Bipolar Disorder (GAGE-BD) project. Bipolar Disord 2023; 25:43–55. 36377516 10.1111/bdi.13271 PMC10265276

[26] Rajashekar N Blumberg HP Villa LM. Neuroimaging studies of brain structure in older adults with bipolar disorder: a review. J Psychiatr Brain Sci 2022; 7:e220006. 36092855 10.20900/jpbs.20220006 PMC9453888

[27▪] Lavin P Rej S Olagunju AT. Essential Data Dimensions for prospective international data collection in Older Age Bipolar Disorder (OABD): recommendations from the GAGE-BD group. Bipolar Disord 2023; [Online ahead of print]. 10.1111/bdi.13312 36843436

[28] Kapczinski NS Mwangi B Cassidy RM. Neuroprogression and illness trajectories in bipolar disorder. Expert Rev Neurother 2017; 17:277–285. 27659841 10.1080/14737175.2017.1240615

[29] Lee Y Lee D Jung H. Heterogeneous early illness courses of Korean patients with bipolar disorders: replication of the staging model. BMC Psychiatry 2022; 22:684. 36333702 10.1186/s12888-022-04318-y PMC9636704

[30] Dols A Korten N Comijs H. The clinical course of late-life bipolar disorder, looking back and forward. Bipolar Disord 2017; 20:459–469. 10.1111/bdi.12586 29227034

[31] Post RM. Transduction of psychosocial stress into the neurobiology of recurrent affective disorder. Am J Psychiatry 1992; 149:999–1010. 1353322 10.1176/ajp.149.8.999

[32] Post RM. Kindling and sensitization as models for affective episode recurrence, cyclicity, and tolerance phenomena. Neurosci Biobehav Rev 2007; 31:858–873. 17555817 10.1016/j.neubiorev.2007.04.003

[33] Goodwin FK Jamison KR. Manic-depressive illness: bipolar disorders and recurrent depression. Oxford: Oxford University Press; 2007.

[34] Berk M Conus P Lucas N. Setting the stage: from prodrome to treatment resistance in bipolar disorder. Bipolar Disord 2007; 9:671–678. 17988356 10.1111/j.1399-5618.2007.00484.x

[35] Kapczinski F Dias VV Kauer-Sant’Anna M. Clinical implications of a staging model for bipolar disorders. Expert Rev Neurother 2009; 9:957–966. 19589046 10.1586/ern.09.31

[36] Orhan M Huijser J Korten N. The influence of social, psychological, and cognitive factors on the clinical course in older patients with bipolar disorder. Int J Geriatr Psychiatry 2021; 36:342–348. 32909298 10.1002/gps.5431

[37] Orhan M Korten N Paans N. Psychiatric symptoms during the COVID-19 outbreak in older adults with bipolar disorder. Int J Geriatr Psychiatry 2021; 36:892–900. 33368692 10.1002/gps.5489

[38] Orhan M Korten N Kok A. The course of psychiatric symptoms in older age bipolar disorder during the COVID-19 pandemic. Int J Bipolar Disord 2022; 10:29. 36472691 10.1186/s40345-022-00274-4 PMC9727013

[39] van der Markt A Beunders AJM Korten NCM. Illness progression in older-age bipolar disorder: exploring the applicability, dispersion, concordance, and associated clinical markers of two staging models for bipolar disorder in an older population. Int J Geriatr Psychiatry 2022; 37: [Epub ahead of print]. 10.1002/gps.5816 PMC9828008 36205029

[40] Sanchez-Moreno J Bonnin CM Gonzalez-Pinto A. Factors associated with poor functional outcome in bipolar disorder: sociodemographic, clinical, and neurocognitive variables. Acta Psychiatr Scand 2018; 138:145–154. 29726004 10.1111/acps.12894

[41] Comes M Rosa A Reinares M. Functional impairment in older adults with bipolar disorder. J Nerv Ment Dis 2017; 205:443–447. 28459727 10.1097/NMD.0000000000000683

[42] Depp CA Davis CE Mittal D. Health-related quality of life and functioning of middle-aged and elderly adults with bipolar disorder. J Clin Psychiatry 2006; 67:215–221. 16566616 10.4088/jcp.v67n0207

[43] Orhan M Korten N Kupka R. Reliability and validity of the functioning assessment short test for older adults with bipolar disorder (FAST-O). Int J Bipolar Disord 2020; 8:28. 33006669 10.1186/s40345-020-00193-2 PMC7532249

[44] Orhan M Millett C Klaus F. Comparing continuous and harmonized measures of depression severity in older adults with bipolar disorder: relationship to functioning. J Affect Disord 2022; 314:44–49. 35803392 10.1016/j.jad.2022.06.074

[45] Paans NP Korten N Orhan M. Is social functioning in older age patients with bipolar disorder associated with affective and/or nonaffective cognition? Int J Geriatr Psychiatry 2022; 37:1–9. 10.1002/gps.5676 34997778

[46] Burdick KE Millett CE. Cognitive heterogeneity is a key predictor of differential functional outcome in patients with bipolar disorder. Eur Neuropsychopharmacol 2021; 53:4–6. 34256309 10.1016/j.euroneuro.2021.06.008 PMC8633033

[47] Vieta E Salagre E Grande I. Early intervention in bipolar disorder. Am J Psychiatry 2018; 175:411–426. 29361850 10.1176/appi.ajp.2017.17090972

[48] Chen MH Li CT Tsai CF. Risk of subsequent dementia among patients with bipolar disorder or major depression: a nationwide longitudinal study in Taiwan. J Am Med Dir Assoc 2015; 16:504–508. 25737262 10.1016/j.jamda.2015.01.084

[49] Diniz BS Teixeira AL Cao F. History of bipolar disorder and the risk of dementia: a systematic review and meta-analysis. Am J Geriatr Psychiatry 2017; 25:357–362. 28161155 10.1016/j.jagp.2016.11.014 PMC5365367

[50] Kessing LV Nilsson FM. Increased risk of developing dementia in patients with major affective disorders compared to patients with other medical illnesses. J Affect Disord 2003; 73:261–269. 12547295 10.1016/s0165-0327(02)00004-6

[51] Wu KY Chang CM Liang HY. Increased risk of developing dementia in patients with bipolar disorder: a nested matched case-control study. Bipolar Disord 2013; 15:787–794. 23992521 10.1111/bdi.12116

[52] Burdick KE Russo M Frangou S. Empirical evidence for discrete neurocognitive subgroups in bipolar disorder: clinical implications. Psychol Med 2014; 44:3083–3096. 25065409 10.1017/S0033291714000439 PMC4797987

[53] Jimenez E Sole B Arias B. Impact of childhood trauma on cognitive profile in bipolar disorder. Bipolar Disord 2017; 19:363–374. 28691361 10.1111/bdi.12514

[54] Kjaerstad HL de Siqueira Rotenberg L Knudsen GM. The longitudinal trajectory of emotion regulation and associated neural activity in patients with bipolar disorder: a prospective fMRI study. Acta Psychiatr Scand 2022; 146:568–582. 36054343 10.1111/acps.13488 PMC9804505

[55] Sole B Jimenez E Torrent C. Cognitive variability in bipolar II disorder: who is cognitively impaired and who is preserved. Bipolar Disord 2016; 18:288–299. 27112120 10.1111/bdi.12385

[56] Ehrlich TJ Ryan KA Burdick KE. Cognitive subgroups and their longitudinal trajectories in bipolar disorder. Acta Psychiatr Scand 2022; 146:240–250. 35690884 10.1111/acps.13460 PMC9545624

[57] Van Rheenen TE Lewandowski KE Bauer IE. Current understandings of the trajectory and emerging correlates of cognitive impairment in bipolar disorder: an overview of evidence. Bipolar Disord 2020; 22:13–27. 31408230 10.1111/bdi.12821

[58] Berk M. Neuroprogression: pathways to progressive brain changes in bipolar disorder. Int J Neuropsychopharmacol 2009; 12:441–445. 18922203 10.1017/S1461145708009498

[59] Lopez-Jaramillo C Lopera-Vasquez J Gallo A. Effects of recurrence on the cognitive performance of patients with bipolar I disorder: implications for relapse prevention and treatment adherence. Bipolar Disord 2010; 12:557–567. 20712758 10.1111/j.1399-5618.2010.00835.x

[60] Moylan S Maes M Wray NR Berk M. The neuroprogressive nature of major depressive disorder: pathways to disease evolution and resistance, and therapeutic implications. Mol Psychiatry 2013; 18:595–606. 22525486 10.1038/mp.2012.33

[61] Martino DJ Samame C Marengo E. A critical overview of the clinical evidence supporting the concept of neuroprogression in bipolar disorder. Psychiatry Res 2016; 235:1–6. 26723135 10.1016/j.psychres.2015.12.012

[62▪] Montejo L Torrent C Jimenez E. Cognition in older adults with bipolar disorder: an ISBD task force systematic review and meta-analysis based on a comprehensive neuropsychological assessment. Bipolar Disord 2022; 24:115–136. 34978124 10.1111/bdi.13175

[63] Montejo L Sole B Jimenez E. Aging in bipolar disorder: cognitive performance and clinical factors based on an adulthood-lifespan perspective. J Affect Disord 2022; 312:292–302. 35752219 10.1016/j.jad.2022.06.030

[64] Orhan M Schouws S van Oppen P. Cognitive functioning in late life affective disorders: comparing older adults with bipolar disorder, late life depression and healthy controls. J Affect Disord 2023; 320:468–473. 36202302 10.1016/j.jad.2022.09.127

[65] Omer E Braw Y Amiaz R Ravona-Springer R. Executive functioning of older adults with bipolar disorder. Int J Geriatr Psychiatry 2021; 36:106–115. 33411378 10.1002/gps.5402

[66] Beunders AJM Kemp T Korten NCM. Cognitive performance in older-age bipolar disorder: investigating psychiatric characteristics, cardiovascular burden and psychotropic medication. Acta Psychiatr Scand 2021; 144:392–406. 34166526 10.1111/acps.13342 PMC8518600

[67] Montejo L Jimenez E Sole B. Identifying neurocognitive heterogeneity in Older Adults with Bipolar Disorder: a cluster analysis. J Affect Disord 2022; 298:522–531. 34788686 10.1016/j.jad.2021.11.028

[68] Stafford J Chung WT Sommerlad A. Psychiatric disorders and risk of subsequent dementia: systematic review and meta-analysis of longitudinal studies. Int J Geriatr Psychiatry 2022; 37:1–22. 10.1002/gps.5711 PMC9325434 35460299

[69] Sinha A Shariq A Said K. Medical comorbidities in bipolar disorder. Curr Psychiatry Rep 2018; 20:36. 29732528 10.1007/s11920-018-0897-8

[70] Firth J Siddiqi N Koyanagi A. The Lancet Psychiatry Commission: a blueprint for protecting physical health in people with mental illness. Lancet Psychiatry 2019; 6:675–712. 31324560 10.1016/S2215-0366(19)30132-4

[71] Crump C Sundquist K Winkleby MA Sundquist J. Comorbidities and mortality in bipolar disorder: a Swedish national cohort study. JAMA Psychiatry 2013; 70:931–939. 23863861 10.1001/jamapsychiatry.2013.1394

[72] Forty L Ulanova A Jones L. Comorbid medical illness in bipolar disorder. Br J Psychiatry 2014; 205:465–472. 25359927 10.1192/bjp.bp.114.152249 PMC4248234

[73] Hayes JF Miles J Walters K. A systematic review and meta-analysis of premature mortality in bipolar affective disorder. Acta Psychiatr Scand 2015; 131:417–425. 25735195 10.1111/acps.12408 PMC4939858

[74] Lambert AM Parretti HM Pearce E. Temporal trends in associations between severe mental illness and risk of cardiovascular disease: a systematic review and meta-analysis. PLoS Med 2022; 19:e1003960. 35439243 10.1371/journal.pmed.1003960 PMC9017899

[75] Osby U Westman J Hallgren J Gissler M. Mortality trends in cardiovascular causes in schizophrenia, bipolar and unipolar mood disorder in Sweden 1987–2010. Eur J Public Health 2016; 26:867–871. 26748100 10.1093/eurpub/ckv245 PMC5054269

[76] Laursen TM. Life expectancy among persons with schizophrenia or bipolar affective disorder. Schizophr Res 2011; 131:101–104. 21741216 10.1016/j.schres.2011.06.008

[77] Laursen TM Wahlbeck K Hallgren J. Life expectancy and death by diseases of the circulatory system in patients with bipolar disorder or schizophrenia in the Nordic countries. PLoS One 2013; 8:e67133. 23826212 10.1371/journal.pone.0067133 PMC3691116

[78] Westman J Hallgren J Wahlbeck K. Cardiovascular mortality in bipolar disorder: a population-based cohort study in Sweden. BMJ Open 2013; 3:1–8. 10.1136/bmjopen-2012-002373 PMC3641504 23604348

[79] Mutz J Young AH Lewis CM. Age-related changes in physiology in individuals with bipolar disorder. J Affect Disord 2022; 296:157–168. 34601303 10.1016/j.jad.2021.09.027

[80] Goldstein BI Schaffer A Wang S Blanco C. Excessive and premature new-onset cardiovascular disease among adults with bipolar disorder in the US NESARC cohort. J Clin Psychiatry 2015; 76:163–169. 25742203 10.4088/JCP.14m09300

[81] Bohman H Agartz I Mansouri S. Preclinical atherosclerosis in adolescents with psychotic or bipolar disorders investigated with carotid high-frequency ultrasound. Brain Behav 2020; 10:e01862. 32997440 10.1002/brb3.1862 PMC7749529

[82] Gimenez-Palomo A Gomes-da-Costa S Dodd S. Does metabolic syndrome or its component factors alter the course of bipolar disorder? A systematic review. Neurosci Biobehav Rev 2022; 132:142–153. 34800584 10.1016/j.neubiorev.2021.11.026

[83] Bora E McIntyre RS Ozerdem A. Neurococognitive and neuroimaging correlates of obesity and components of metabolic syndrome in bipolar disorder: a systematic review. Psychol Med 2019; 49:738–749. 30326979 10.1017/S0033291718003008

[84] Warner A Holland C Lobban F. Physical health comorbidities in older adults with bipolar disorder: a systematic review. J Affect Disord 2023; 326:232–242. 36709829 10.1016/j.jad.2023.01.083

[85] Beunders AJM Kok AAL Kosmas PC. Physical comorbidity in Older-Age Bipolar Disorder (OABD) compared to the general population: a 3-year longitudinal prospective cohort study. J Affect Disord 2021; 288:83–91. 33845328 10.1016/j.jad.2021.03.057

[86▪] Almeida OP Dols A Blanken M. Physical health burden among older men and women with bipolar disorder: results from the Gage-Bd Collaboration. Am J Geriatr Psychiatry 2022; 30:727–732. 34980553 10.1016/j.jagp.2021.12.006

[87] Correll CU Detraux J De Lepeleire J De Hert M. Effects of antipsychotics, antidepressants and mood stabilizers on risk for physical diseases in people with schizophrenia, depression and bipolar disorder. World Psychiatry 2015; 14:119–136. 26043321 10.1002/wps.20204 PMC4471960

[88] Abosi O Lopes S Schmitz S Fiedorowicz JG. Cardiometabolic effects of psychotropic medications. Horm Mol Biol Clin Investig 2018; 36:1–27. 10.1515/hmbci-2017-0065 PMC6818518 29320364

[89] Xu N Huggon B Saunders KEA. Cognitive impairment in patients with bipolar disorder: impact of pharmacological treatment. CNS Drugs 2020; 34:29–46. 31808104 10.1007/s40263-019-00688-2

[90] Yatham LN Kennedy SH Parikh SV. Canadian Network for Mood and Anxiety Treatments (CANMAT) and International Society for Bipolar Disorders (ISBD) 2018 guidelines for the management of patients with bipolar disorder. Bipolar Disord 2018; 20:97–170. 29536616 10.1111/bdi.12609 PMC5947163

[91] Shulman KI Almeida OP Herrmann N. Delphi survey of maintenance lithium treatment in older adults with bipolar disorder: an ISBD task force report. Bipolar Disord 2019; 21:117–123. 30375703 10.1111/bdi.12714 PMC6587471

[92] Rej S Herrmann N Gruneir A. Association of lithium use and a higher serum concentration of lithium with the risk of declining renal function in older adults: a population-based cohort study. J Clin Psychiatry 2020; 81:1–8. 10.4088/JCP.19m13045 32841553

[93] Fotso Soh J Klil-Drori S Rej S. Using lithium in older age bipolar disorder: special considerations. Drugs Aging 2019; 36:147–154. 30613911 10.1007/s40266-018-0628-1

[94] Shulman KI Sajatovic M Dols A. Laboratories should provide a separate therapeutic range for serum lithium levels in maintenance treatment of older adults with bipolar disorder (OABD). Bipolar Disord 2019; 21:190–191. 30861253 10.1111/bdi.12769

[95] Araldi E Jutzeler CR Ristow M. Lithium treatment extends human lifespan: findings from the UK Biobank. Aging (Albany NY) 2023; 15:421–440. 36640269 10.18632/aging.204476 PMC9925675

[96▪] Forlenza OV Hajek T Almeida OP. Demographic and clinical characteristics of lithium-treated older adults with bipolar disorder. Acta Psychiatr Scand 2022; 146:442–455. 35837985 10.1111/acps.13474 PMC9588573

[97] Flapper M van Melick E van Campen J. Tolerability of lithium: a naturalistic discontinuation study in older inpatients (>/=60 years). Int J Geriatr Psychiatry 2021; 36:1231–1240. 33644915 10.1002/gps.5517

[98▪] Chen P Eyler LT Gildengers A. Demographic and clinical characteristics of antipsychotic drug-treated older adults with bipolar disorder from the Global Aging & Geriatric Experiments in Bipolar Disorder Database (GAGE-BD). Psychopharmacol Bull 2022; 52:8–33. 10.64719/pb.4431 PMC9172555 35721813

[99] Olagunju AT Morgan JA Aftab A. A review of the evidence base for nutrition and nutritional supplements in older adults with bipolar disorder: a report from the OABD task force. J Frailty Aging 2021; 10:241–246. 34105708 10.14283/jfa.2020.64 PMC8715337

